# Supplementary material for: The interactome of intact mitochondria by cross-linking mass spectrometry provides evidence for coexisting respiratory supercomplexes
Source: Mol Cell Proteomics. 2017 Dec 8;17(2):216–32. doi: 10.1074/mcp.RA117.000470 (PMC5795388; doi:10.1074/mcp.RA117.000470)
Supplement: Supplemental Data [file supp_RA117.000470_133922_0_supp_23978_fzffwf.zip › spectra_annotation/mito_spectra_annotation/261-1-3-1-12-1.pdf]

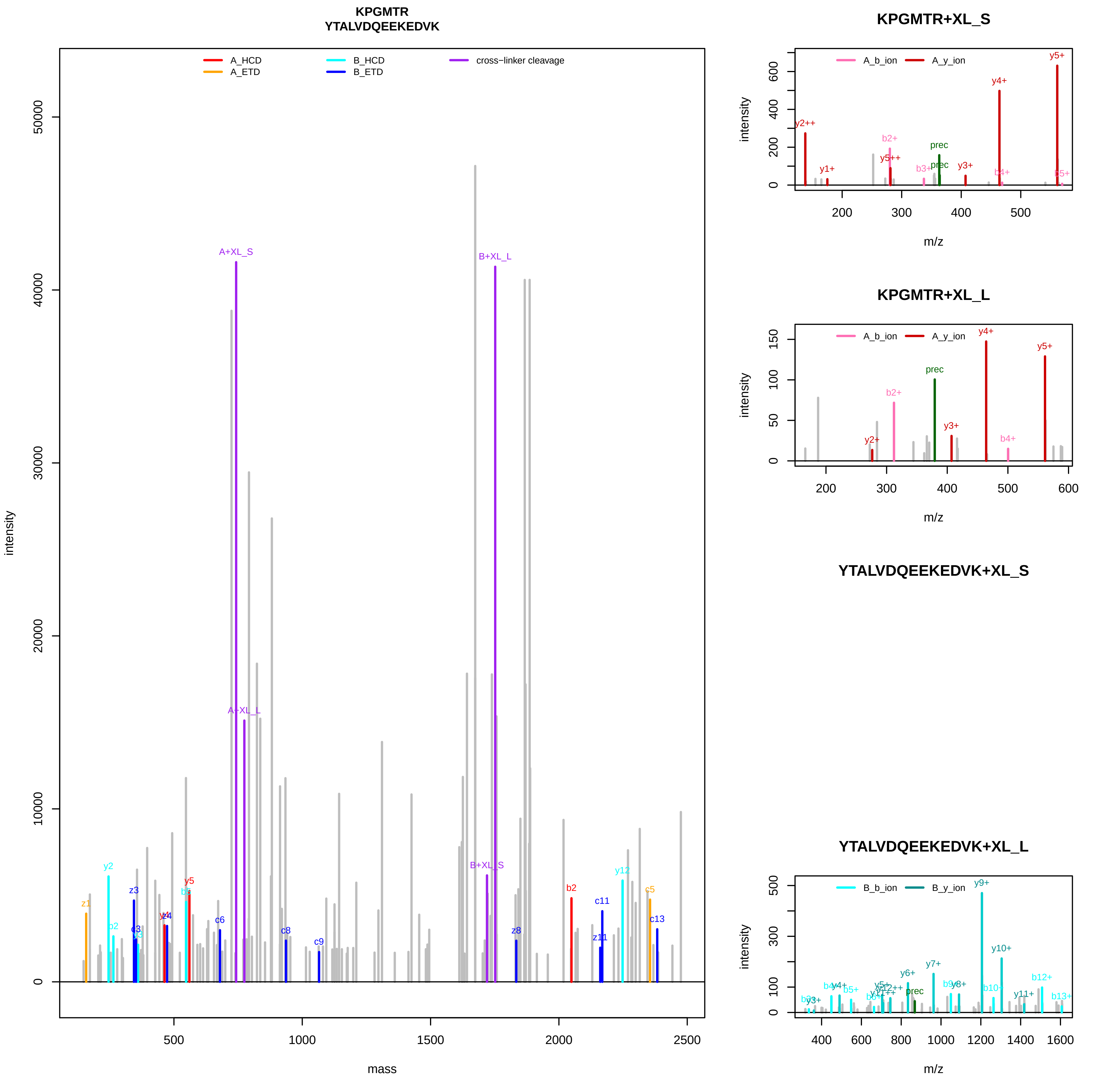
KPGMTR
YTALVDQEEKEDVK
A_HCD
A_ETD
B_HCD
B_ETD
cross−linker cleavage
intensity
mass
0
10000
20000
30000
40000
50000
500
1000
1500
2000
2500
A+XL_S
B+XL_L
A+XL_L
B+XL_S
z1
y2
b2
z3
c3
y3
y4
z4
b5
y5
c6
c8
c9
z8
b2
c11
z11
y12
c5
c13
KPGMTR+XL_S
A_b_ion
A_y_ion
y5+
y4+
y2++
y1+
b2+
y5++
prec
b3+
y3+
b4+
b5+
m/z
KPGMTR+XL_L
y2+
y3+
b4+
YTALVDQEEKEDVK+XL_S
YTALVDQEEKEDVK+XL_L
B_b_ion
B_y_ion
y9+
y10+
y7+
y6+
b12+
b13+
y11+
b10+
y8+
b9+
y4+
b4+
b5+
y3+
b6+
y5+
y12++
y11++
400
600
800
1000
1200
1400
1600
